# Telemedicine in Primary Care for Patients With Chronic Conditions: The ValCrònic Quasi-Experimental Study

**DOI:** 10.2196/jmir.7677

**Published:** 2017-12-15

**Authors:** Domingo Orozco-Beltran, Manuel Sánchez-Molla, Julio Jesus Sanchez, José Joaquin Mira, ValCrònic Research Group

**Affiliations:** ^1^ Catedra de Medicina de Familia Department of Clinical Medicine Miguel Hernandez University San Juan de Alicante Spain; ^2^ San Juan de Alicante Hospital San Juan Health District San Juan de Alicante Spain; ^3^ Elche Health Department Hospital General de Elche Elche Spain; ^4^ Ingeniería y Proyectos e-Health Telefónica Spain Madrid Spain; ^5^ Alicante Sant Joan Health District Alicante Spain; ^6^ Health Psychology Department Miguel Hernandez University San Juan de Alicante Spain; ^7^ Ministry of Health of Valencia Valencia Spain

**Keywords:** chronic disease, primary health care, telemedicine

## Abstract

**Background:**

The increase of chronic diseases prevalence has created the need to adapt care models and to provide greater home supervision.

**Objective:**

The objective of our study was to evaluate the impact of telemonitoring on patients with long-term conditions at high risk for rehospitalization or an emergency department visit, in terms of target disease control (diabetes, hypertension, heart failure, and chronic obstructive pulmonary disease).

**Methods:**

We conducted a quasi-experimental study with a before-and-after analysis to assess the effectiveness of the ValCrònic program after 1 year of primary care monitoring. The study included high-risk patients with 1 or more of the following conditions: diabetes, high blood pressure, heart failure, and chronic obstructive pulmonary disease. We assessed risk according to the Community Assessment Risk Screen. Participants used an electronic device (tablet) to self-report relevant health information, which was then automatically entered into their eHealth record for consultation.

**Results:**

The total sample size was 521 patients. Compared with the preintervention year, there were significant reductions in weight (82.3 kg before vs 80.1 kg after; *P*=.001) and in the proportion of people with high systolic (≥140 mmHg; 190, 36.5% vs 170, 32.6%; *P*=.001) and diastolic (≥90 mmHg; 72, 13.8% vs 40, 7.7%; *P*=.01) blood pressures, and hemoglobin A_1c_ ≥8% (186, 35.7% vs 104, 20.0%; *P*=.001). There was also a decrease in the proportion of participants who used emergency services in primary care (68, 13.1% vs 33, 6.3%; *P*<.001) and in hospital (98, 18.8% vs 67, 12.8%; *P*<.001). Likewise, fewer participants required hospital admission due to an emergency (105, 20.2% vs 71, 13.6%; *P*<.001) or disease exacerbation (55, 10.5% vs 42, 8.1%; *P*<.001).

**Conclusions:**

The ValCrònic telemonitoring program in patients at high risk for rehospitalization or an emergency department visit appears to be useful to improve target disease control and to reduce the use of resources.

## Introduction

Demographic and epidemiological patterns are changing with aging populations and increased prevalence of chronic diseases, causing reduced mobility, along with a need to adapt care models and provide greater home supervision [1,2].

A 2008 review by the Canadian Agency for Drugs and Technologies in Health [3] described the high prevalence of chronic diseases, the great financial and social costs involved, and the attractive prospect of possibly improving patient care through telemedicine. Home telemonitoring and telephone support are the most frequently used ways to perform this kind of monitoring in chronic disease, and there is an important distinction to be made between synchronous (real-time) and asynchronous telemonitoring.

Noncommunicable diseases, particularly cardiovascular diseases, diabetes, cancer, and chronic respiratory diseases, are responsible for more deaths globally than all other causes combined [4]. Many of these conditions, including diabetes, high blood pressure, heart failure, and chronic obstructive pulmonary disease (COPD), can be managed through home telemonitoring programs, which enable health care professionals to monitor patients’ progress and to preempt relapses by using information on vital signs and remote symptom questionnaires [5-8].

With regard to comorbid chronic diseases, telemedicine may decrease the use of resources and mortality compared with standard care [9], although there is no evidence that it improves quality of life or satisfaction.

Most studies [5-9] have reported benefits of telemedicine for patients with hypertension, diabetes, heart failure, or COPD with controversial results. It is difficult to pool intervention types and to define what telemonitoring entails. Some authors considered that telephone call monitoring performed by nurses qualifies as home telemonitoring [10], while other authors disagreed [11,12]. A systematic review of home telemonitoring for COPD by Bolton et al found methodological limitations, and those authors recommended improving and expanding studies and considering the costs [13].

In Spain, most projects for monitoring diseases are in hospital settings, and they are associated with a high rate of readmissions. A recent experience with home cardiac rehabilitation after a coronary event [14] showed that a telemonitoring program appears to be useful for improving the risk profile in acute coronary syndrome survivors and can be an effective tool for secondary prevention. A 3-country project on telemedicine for cardiopulmonary rehabilitation in people with COPD [15] found that integrated care services supported by information and communication technologies can improve COPD management. Primary care experiences such as the TELBIL study in the Basque Country in Spain are rare. That program involved participants at very high risk of heart failure and COPD and reported very encouraging results, including reduced hospital admissions, length of hospital stay, and emergency visits [16]. The PROMETE study showed similar results in patients with severe COPD [17]. Both studies had very few participants. However, their conditions were very serious and generally led to many emergencies and hospital readmissions.

Although previous literature shows that many telemedicine programs have been implemented and evaluated with favorable effects, most of the studies included patients with 1 specific chronic condition, and these study samples are not broadly representative of patients encountered in everyday practice [18]. To assess a telemonitoring program in the real-world population, it is necessary that the study sample include patients encountered in routine clinical practice settings. In addition, high-risk patients are often managed by primary and specialty care, so both hospital and primary care settings should be involved in telemonitoring studies that address such patients.

The aim of this study was to evaluate the impact of telemonitoring on patients with 1 or more long-term conditions at high risk for rehospitalization or an emergency department visit, in terms of target disease control (diabetes, high blood pressure, heart failure, and COPD).

## Methods

### Study Design

This was a before-and-after quasi-experimental intervention study.

### Setting and Study Period

ValCrònic was a 5-year (2011-2016) telemonitoring program in 4 Spanish health centers in the Valencia Region (population 5 million), situated on the Mediterranean coast, within the health departments of Sagunto and Elche: Sagunto health center, Sagunto Port health center II, Elx-El Raval health center, and Santa Pola health center. In December 2013, two additional health centers were incorporated: Elx-Altabix health center and Elx-San Fermin health center. The program was led by primary care services in collaboration with the referral hospital and other institutions in the health sector, with the participation of more than 150 professionals. In the Spanish public health system, primary health centers from the same area have the same referral hospital where patients are admitted. Since the participating health centers belonged to 2 different areas, 2 referral hospitals collaborated in the study in order to collect all hospital admissions and emergency department visits.

Development of the technical procedures and protocols began in April 2011, and participants were recruited from February 2012 to February 2015 for a 1-year telemonitoring intervention. We compared the clinical outcomes before and after the intervention. The program ended in February 2016.

### Study Sample

The study included people at high risk for rehospitalization or an emergency department visit with 1 or more of the following conditions: heart failure, COPD, type 2 diabetes mellitus, and arterial hypertension. Heart failure and COPD are the most frequent causes of nonscheduled hospital admissions, while diabetes and hypertension consume a large amount of health care resources in the field of primary care due to their high prevalence. Furthermore, we selected these chronic diseases because patients can easily measure their indicators, and which were also sensitive to changes introduced as part of the intervention. We excluded patients who did not sign the informed consent form, did not have a telephone, or had a life expectancy of less than 1 year (based on their physician’s opinion).

The Kaiser Permanente model [19] recommends stratifying the risk of patients for hospitalizations or emergency department encounters according to the Community Assessment Risk Screen (CARS) [20]; this screen can identify high-risk patients and has been validated in the Valencia Region by the Polibienestar Research Institute at the University of Valencia [21]. To improve the validity of the scale, we complemented the result obtained (high risk or not) with the clinical judgement of the participant’s usual physician.

We used a consecutive, nonprobability sampling method in the primary care setting. In addition, we actively recruited patients who met the inclusion criteria (according to the data in their eHealth records), by means of written invitations to participate in the program.

### Intervention and Measurements

The ValCrònic program is preventive and based on innovative approaches to chronicity such as the chronic care model [22] and the Kaiser Permanente model [19]. The program features continuous telemonitoring of patients with chronic conditions and multiple comorbidities. It is led mainly by primary care but operates in collaboration with hospital services (especially the general medicine service, among others), primary care- and hospital-based emergency services, and the home hospitalization unit.

The intervention consisted of several components: participants measured their own vital signs related to the conditions included in the study and automatically entered them into their eHealth record via a wireless electronic device (tablet) provided to them for the study. In addition, an automated alert system was set up to promptly detect any alterations, which promoted health professionals’ proactive involvement. Participants also received health education and awareness interventions tailored to their conditions through informative videos available in the tablet.

On the basis of different possible combinations of the 4 diseases included in the program, we defined 8 individualized care programs and monitored each condition from the patient’s home, based on the following indicators: (1) heart failure (blood pressure, heart rate, symptom questionnaire, and weight gain), (2) COPD (COPD Assessment Test [CAT] questionnaire [22], oxygen saturation), (3) heart failure plus COPD, (4) heart failure plus diabetes (capillary glycemia), (5) diabetes plus COPD, (6) COPD plus arterial hypertension (blood pressure), (7) heart failure plus COPD plus diabetes, and (8) diabetes plus arterial hypertension.

**Figure 1:**
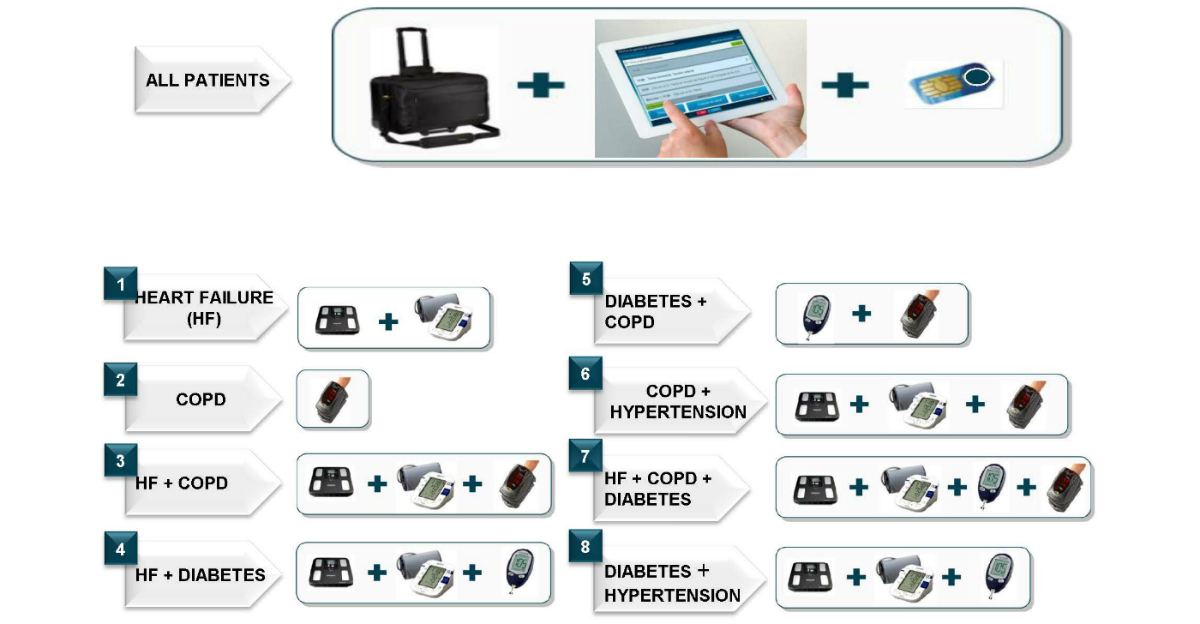
Telemonitoring program devices, according to a patient’s disease: scales, blood pressure monitor, glucometer, and pulse oximeter. COPD: chronic obstructive pulmonary disease.

**Figure 2:**
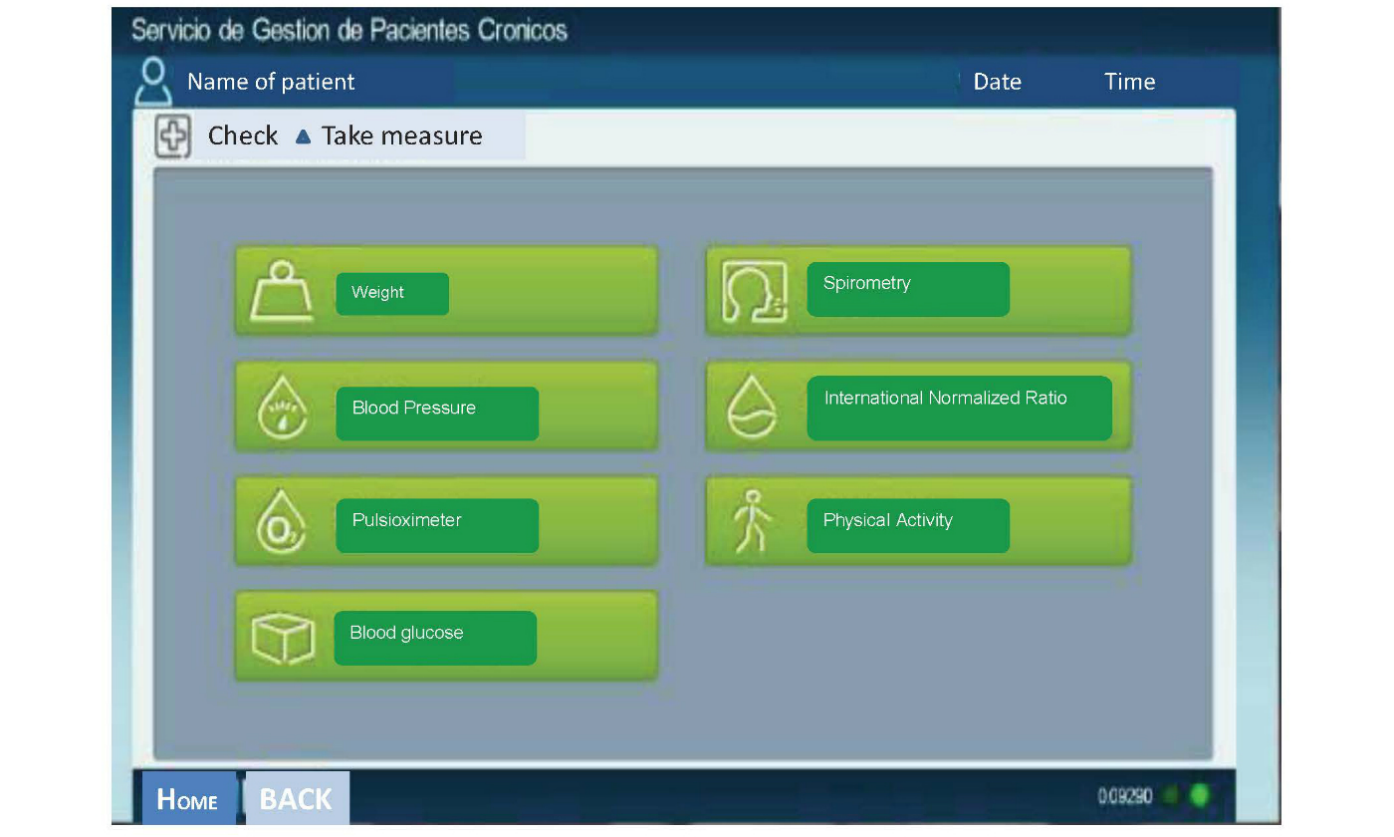
Interface display on the tablet.

Participants received different tools for self-measurement according to their diseases (Figure 1), along with videos instructing them in how to use them: a set of scales, a blood pressure monitor, a glucometer, and a pulse oximeter. In addition, we used health questionnaires for COPD and heart failure. Participants with COPD also completed the CAT questionnaire [23]. All information was recorded and automatically sent to the health center using the eHealth record functionalities included on the tablet (Figure 2). The frequency of the self-measurements was established individually with each patient, but health information was usually submitted on a weekly basis.

The self-reports were entered in the eHealth records in a similar way to how they would be entered in face-to-face visits to the center. This was possible due to the existence of a unique patient identifier and eHealth record for each patient within the Abucasis health information system (version II; Valencia Health Agency), which was used by the professionals in primary and specialized care in this study. Several months of prior technical work was necessary to ensure compatibility between the data sent from patients’ homes and the eHealth records. Changes were also implemented in the clinical records used for hospital admissions in every health department to enable identification of ValCrònic patients in the event they went to an emergency department or were admitted to hospital.

Alerts were programmed into the system to automatically display self-measurement values outside the normal range established for each participant. The primary care nurse who was usually in charge of managing the patient’s care was responsible for following up on these alerts by means of the relevant action protocol. Every day, when the nurse opened the eHealth record app, they received information about the patients for whom an alert had been raised in the previous 24 hours or over the weekend. In the event of an emergency, the standard face-to-face procedure was followed.

Upon receipt of the self-measurements in the eHealth center, it was up to the nurse to decide whether to call participants to check the values, ask them to come to the health center, go to their home, or consult a doctor. Before program launch, all the doctors, nurses, and administrative staff at the participating centers received specific training on the study process, the program, and the devices.

In addition, all participants received group training from their primary care nurses or doctors regarding the use of the monitoring devices and software apps. This training included content aimed at improving patient knowledge of self-care. In addition, participants received individualized training in their homes from technical personnel regarding the use of telemedicine devices. Patients received the equipment in their homes and had access to technical assistance via telephone or in-person visits from the company in charge of managing the operation of the hardware and software (eHealth department of Telefónica España, SA, Madrid, Spain). The communication protocols between monitoring devices, tablets, and eHealth records were developed and implemented jointly by the eHealth center, Abucasis, and Telefónica eHealth technicians.

A senior management committee of 9 people was set up to monitor the activities of the ValCrònic program, composed of representatives from the Regional Health Ministry, including the General Health Care Directorate, the Health Area Directorate, the 2 managers of the participating health departments (Sagunto and Elx), the head of Information Technology Systems, and the technical program coordinator; and representatives of Telefónica, who were in charge of the telemedicine devices used. Meetings were held on a quarterly basis. A scientific committee was also set up, composed of the technical program coordinator, representatives of the professionals of the 2 participating health departments, and representatives of Telefónica.

### Outcomes

We recorded the weight and heart rate of patients, the proportion of patients with poor control of systolic (≥140 mmHg) or diastolic (≥ 90 mmHg) blood pressure, and the proportion of patients with poor control of hemoglobin A_1c_ (HbA_1c_) (≥8%). We also recorded visits to primary care- or hospital-based emergency services due to an exacerbation of the target diseases. Finally, we recorded unscheduled (emergency) hospital admissions. In addition, we calculated indicators of clinical relevance to the ValCrònic program: absolute risk reduction, relative risk reduction, and number needed to treat to prevent a harmful outcome.

### Variables

We collected demographic data (age, sex) from all the participants, as well as the indicators for each condition: weight (kg), heart rate (beats/min), blood pressure (mmHg), capillary glycemia (mg/dL), and HbA_1c_ from a venous blood sample (%). Although other variables were collected during the study period, we did not include them, such as the results of the questionnaires regarding signs and symptoms, in the analyses reported in this paper.

### Data Collection Method

All the information was recorded in the eHealth records either on-site or remotely from the self-measurements made by patients in their homes. We followed and monitored all participants for 1 year to manage their disease.

### Statistical Analysis

#### Sample Size

We performed a power calculation to detect a difference of 10% in the proportion of patients who required urgent hospital admissions (unscheduled), estimating 20% in the control group (ie, in the participants before the intervention) and 10% in the intervention group. We determined that we needed a sample size of 174 participants for a power of 80% (20% beta risk) and a confidence level of 95% (5% alpha risk), including possible attrition of 10%.

#### Analytical Strategy

We conducted a before-and-after analysis to assess the impact of the intervention on the control of blood pressure and HbA_1c_, as well as on visits to emergency services and hospital admissions. We compared means and proportions of the first visit (preintervention) with the last visit (postintervention) using the IBM SPSS PC version 21 statistical package (IBM Corporation). To assess intervention effects, we used the *t* test and chi-square statistical test. We calculated indicators of clinical relevance to the ValCrònic program, absolute risk reduction, relative risk reduction, and number needed to treat to prevent a harmful outcome.

### Ethical Aspects

The Committee of Ethics and Clinical Research at the Elx Health Department approved this study. We complied with the procedures and regulations of Law 15/1999 on the Protection of Personal Data of December 13, 1999 (Spanish Data Protection Agency). All participants signed a statement of informed consent and committed to taking care of the equipment loaned to them.

## Results

**Figure 3:**
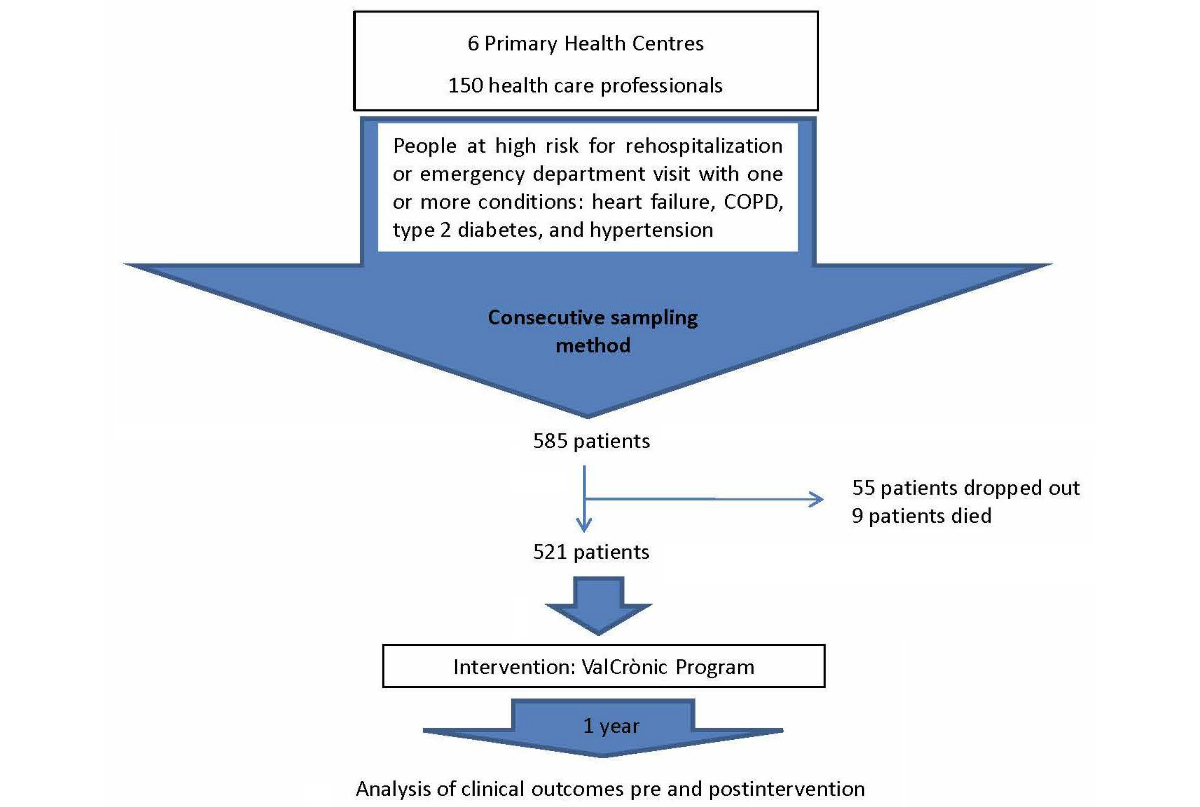
Study flowchart. COPD: chronic obstructive pulmonary disease.

We invited 585 patients to participate, 55 of whom dropped out of the program because they found it difficult to take the measurements alone and had no help. A total of 9 participants died during the intervention. Thus, we included 521 participants in the analysis (Figure 3). Participants’ average age was 70.4 years, and over half (n=318, 61.0%) were men. The 70- to 79-year age group was the largest, amounting to 32.3% (n=168) of the total. With regard to hospital admissions, 28.2% (n=147) of the participants were admitted at some point during the year of study. The average number of drug prescriptions was 8.3 per participant. The most prevalent mix of comorbidities was hypertension and diabetes, which involved more than one-third of patients, followed by COPD plus hypertension, and heart failure plus hypertension plus diabetes. These 3 combinations accounted for 61.0% of patients (Table 1).

**Figure 4:**
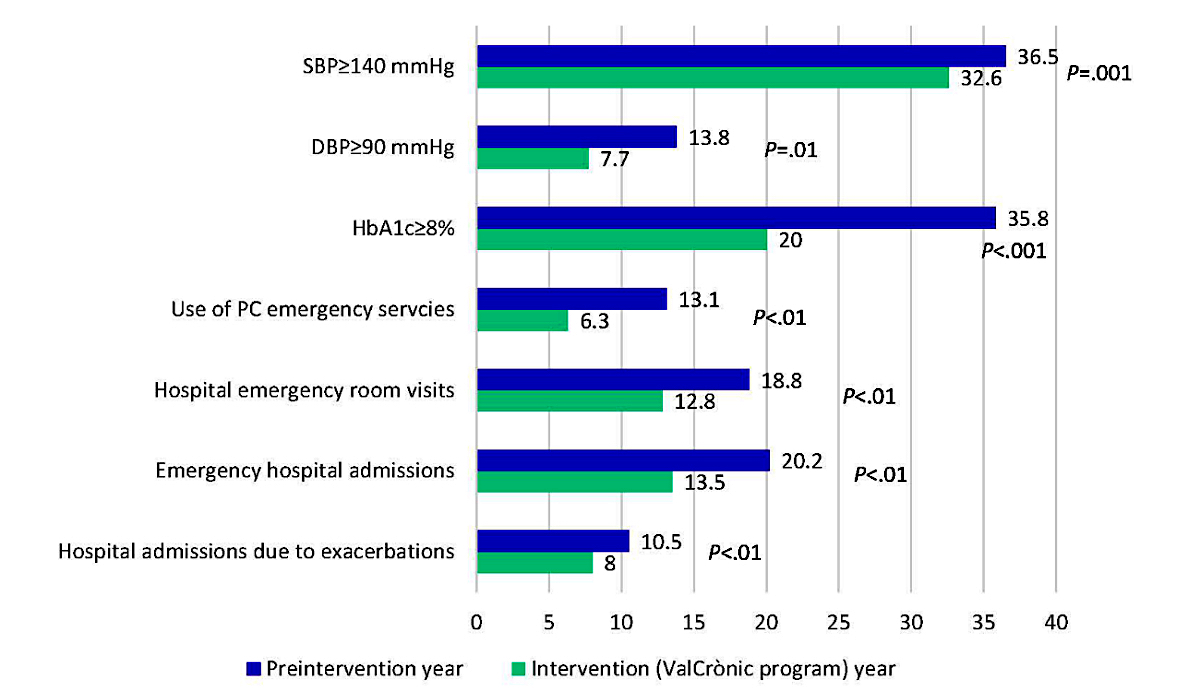
Comparison between preintervention year and intervention (ValCrònic program) year regarding study outcomes: proportion of patients with poor blood pressure and hemoglobin A_1c_ (HbA_1c_) control; and proportion of patients who visited primary care- (PC) or hospital-based emergency services due to an exacerbation or were hospitalized. DBP: diastolic blood pressure; SBP: systolic blood pressure.

The ValCrònic program had a significant impact on weight (82.3 kg before vs 80.1 kg after; *P*=.001) and heart rate (74.2 beats/min vs 71.3 beats/min; *P*=.08) in participants in the intervention compared with the preintervention year. Likewise, participation was associated with better control of hypertension: the proportion of people with high systolic (≥140 mmHg) and diastolic (≥90 mmHg) blood pressures fell by 10% and 44%, respectively. The proportion of people with HbA_1c_ of 8% or more also decreased significantly, by 44% (Figure 4).

Figure 4 shows the proportion of participants who went to primary care- or hospital-based emergency services in the year before and the year of the intervention due to disease exacerbation. The ValCrònic program significantly reduced the proportion of people who needed emergency services.

In addition, during the ValCrònic year, the proportion of participants who required hospital admissions, either as a result of an emergency or due to a disease exacerbation, was significantly reduced (Figure 4).

Table 2 shows the indicators that were of clinical relevance to the ValCrònic program.

**Table 1:** Participant characteristics (n=521).

Characteristics		Data
**Sex, n (%)**		
	Female	203 (38.9)
	Male	318 (61.1)
Age (years), mean (SD), 95% CI		70.4 (10.3), 68.9-71.9
**Hospital admissions during the year, n (%)**		
	0	374 (71.7)
	≥1	147 (28.3)
No. of drugs prescribed, mean (SD), 95% CI		8.25 (4.0), 7.65-8.85
**Disease type**^a^**, n**		
	Heart failure	182
	COPD^b^	178
	Diabetes	333
	Arterial hypertension	396
**No. of conditions, n**		
	1	9
	2	347
	3	141
	4	24

^a^Participants could have more than 1 disease.

^b^COPD: chronic obstructive pulmonary disease.

**Table 2:** Participants with outcomes of clinical relevance before and during the intervention (n=521).

Outcomes	Time point, n (%)		ARR^a^ (95% CI)	RRR^b^ (95% CI)	NNT^c^ (95% CI)
	Preintervention year	ValCrònic year			
Systolic blood pressure ≥140 mmHg	190 (36.5)	170 (32.6)	3.9 (0-10)	10.7 (0-25)	26 (10-52)
Diastolic blood pressure ≥90 mmHg	72 (13.8)	40 (7.7)	6.1 (2-10)	44.2 (20-62)	16 (9.9-40.3)
Hemoglobin A_1c_≥8%	186 (35.7)	104 (20.0)	15.8 (10-21)	44.1 (31-55)	6 (4.7-9.6)
Use of primary care emergency services due to exacerbation of ValCrònic conditions	68 (13.1)	33 (6.3)	6.8 (3-10)	51.9 (29-68)	15 (9.5-30.8)
Visit to hospital emergency due to exacerbation of ValCrònic conditions	98 (18.8)	67 (12.8)	6.1 (2-11)	32.2 (9-49)	16 (9.5-62.9)
Emergency hospital admission(s)	105 (20.2)	71 (13.6)	6.7 (2-11)	33.2 (11-49)	15 (8.9-48.1)
Hospital admission(s) due to exacerbation of ValCrònic conditions	55 (10.5)	42 (8.1)	2.5 (0-8)	23.8 (9-37)	40 (20-58)

^a^ARR: absolute risk reduction.

^b^RRR: relative risk reduction.

^c^NNT: number needed to treat to prevent a harmful outcome.

## Discussion

### Principal Findings

Compared with the preintervention year, during the intervention year, people with at least one of 4 chronic diseases (hypertension, diabetes mellitus, COPD, or heart failure) who participated in the ValCrònic program had better weight, heart rate, blood pressure, and glycemic control. In addition, primary care emergency and hospital emergency visits were decreased, despite the participants being a year older and a year further along in their disease evolution.

### Comparison With Prior Work

In participants with diabetes, participation in ValCrònic was associated with improved disease control, reducing the proportion of patients with HbA_1c_ ≥8% by 44% in the year of monitoring. Similarly, previous telemonitoring studies in patients with diabetes reported improved control of HbA_1c_ and fewer admissions, although with more visits to a doctor (primary care or specialist) [6,9].

The ValCrònic program also reduced the proportion of participants with poorly controlled systolic and diastolic blood pressures, by 10% and 44%, respectively. A review by Verberk et al [5] found that participants receiving telemedicine experienced a greater reduction in blood pressure than the usual-care control group. As for the use of health care resources, ValCrònic led to a 51.9% reduction in visits to the primary care emergency department and a 32.3% reduction in visits to the hospital emergency department. Emergency admissions fell by 33.2% and admissions due to worsening of the conditions being monitored fell by 23%. The literature contains encouraging examples of studies reporting that telemonitoring resulted in reduced use of sociohealth resources [24] and reduced mortality (by 24%) and readmissions (by 28%) in patients with heart failure, especially for New York Heart Association classes III and IV [25]. Telephone support only reduced mortality due to relapses, but not overall mortality, while heart rate monitoring decreased the risk of hospital admissions due to heart failure by 43% [25]. Visits to emergency services also decreased, but contact with primary care increased, and patient satisfaction and quality of life improved [7,24]. In COPD, most studies have been in people aged over 65 years and with forced expiratory volume in the first second of expiration of 27% to 43% (Global Initiative for Chronic Obstructive Lung Disease class 2-4). Although those authors reported reductions in readmissions and emergency visits, there were no differences in mortality rate, quality of life, or satisfaction [8,26,27].

The Whole Systems Demonstrator, which started in 2008, is the largest telemonitoring experience in Europe, involving 3230 patients in 179 primary care groups. Investigators have found a reduction in mortality and secondary resource consumption in telemonitored patients, but at a higher cost [28]. Giamouzis et al emphasized the need to define the profile of the patient who can benefit from the intervention and for how long [29]. ValCrònic did not enlist additional health care staff to run the program, and we included participants at high risk, often with several comorbidities, as occurs with this type of patient with chronic diseases. Unlike previous studies, this study did not focus exclusively on 1 specific disease, making this program more pragmatic and more generalizable to the realities of clinical practice.

The clinical benefits obtained show that telemonitoring of patients provides an additional benefit to the medical and nonmedical measures used to date, and thus is another option for treating patients with chronic disease. Table 2 shows that the number needed to treat associated with the program are very favorable compared with the results of other common health interventions. EMPA-REG investigators found that it was necessary to treat 39 patients with empagliflozin to prevent 1 cardiovascular event [30]; in the LEADER study, 66 patients treated with liraglutide were needed to prevent 1 major cardiovascular event [31]; and in the SUSTAIN-6 study, 45 patients treated with semaglutide were needed [32]. To prevent 1 death in the 4S study, 30 patients treated with simvastatin during 5.4 years were needed [33], and when using ramipril, 56 patients treated for 5 years were needed [34]. Thus, ValCrònic is well within the normally accepted parameters for implementation in clinical practice [35].

Regarding the opinion of the participants, a previously published report described high satisfaction among ValCrònic participants [36]. We also highlight the peace of mind reported by patients and their families, stemming from the knowledge that health professionals were remotely monitoring any changes in glycemia, blood pressure, and oxygen saturation, and that they would follow up if necessary.

### Limitations

The limitations of this study are mainly methodological. We dichotomized participant risk (high risk or not) using the CARS scale and, to improve its validity, we complemented the result obtained with the clinical judgement of the professional who usually treated the patient. There are numerous risk assessment scales, but their validity is usually limited, so we believed it was important that health care professionals assisted in the final selection based on their clinical experience and knowledge of the patient’s sociohealth environment.

It is possible that included participants would have been more predisposed to the use of telemedicine. In any case, the high number of participants included suggests that many elderly patients or their family members feel comfortable handling these devices. Elsewhere [36], our group described the degree of satisfaction of patients and their families with the ValCrònic program. We also conducted a survey in patients who opted not to participate, finding no substantial differences in overall satisfaction, although for very different reasons, not only a disinclination to use the health monitoring devices.

As we used a before-and-after study design, we lacked a parallel control group, and the results of this study have to be interpreted with caution. Secular trends or sudden changes might have made it difficult to attribute observed changes to the intervention. However, since it was carried out in two specific health areas, information regarding potential interventions carried out outside the program is available, and there was no interference in this regard. The fact that participants’ diseases had evolved for an additional year, and that they were a year older at study end, adds even more value to the good results obtained.

Another factor to take into account is the Hawthorne effect, or the feeling of being observed. In all intervention studies, there is a potential additional beneficial effect due to people knowing that they are participating in a study. However, given the high risk in the included participants, with more than 20% of them having been hospitalized in the previous year, it is difficult to imagine that a benefit like the one described could occur purely as a result of the Hawthorne effect.

Without a clear understanding of the economic implications of telemonitoring interventions, it will be difficult to establish informed national policies regarding reimbursement for these programs. Cost-effectiveness analyses of this program will be published.

### Conclusions

Compared with the preintervention year, during the intervention year, people with at least one of 4 chronic diseases (hypertension, diabetes mellitus, COPD, or heart failure) at high risk for rehospitalization or an emergency department visit and who participated in the ValCrònic telemonitoring program had better weight control, reduced blood pressure and glycemia, and made fewer visits to primary care- or hospital-based emergency services due to disease exacerbation. In addition, hospitalizations due to an exacerbation of a chronic disease decreased.

## Abbreviations

CARS: Community Assessment Risk Screen
CAT: COPD Assessment Test
COPD: chronic obstructive pulmonary disease
HbA_1c_: hemoglobin A_1c_
